# Tool Wear and Milling Characteristics for Hybrid Additive Manufacturing Combining Laser Powder Bed Fusion and In Situ High-Speed Milling

**DOI:** 10.3390/ma15031236

**Published:** 2022-02-07

**Authors:** David Sommer, Dominik Pape, Cemal Esen, Ralf Hellmann

**Affiliations:** 1 Applied Laser and Photonics Group, University of Applied Sciences Aschaffenburg, 63743 Aschaffenburg, Germany; domipap@web.de (D.P.); ralf.hellmann@th-ab.de (R.H.); 2 Applied Laser Technologies, Ruhr-University Bochum, 44801 Bochum, Germany; esen@lat.rub.de

**Keywords:** hybrid additive manufacturing, high-speed milling, laser powder bed fusion, tool wear

## Abstract

We report on milling and tool wear characteristics of hybrid additive manufacturing comprising laser powder bed fusion and in situ high-speed milling, a particular process in which the cutter mills inside the powder bed without any cooling lubricant being applicable. Flank wear is found to be the dominant wear characteristic with its temporal evolution over utilization period revealing the typical s-shaped dependence. The flank wear land width is measured by microscopy and correlated to the achievable surface roughness of milled 3D-printed parts, showing that for flank wear levels up to 100 μm a superior surface roughness below 3 μm is accessible for hybrid additive manufacturing. Further, based on this correlation recommended tool, life scenarios can be deduced. In addition, by optimizing the finishing tool start position and the number of afore-built layers, the milling process is improved with respect to the maximum millable angle for undercut surfaces of 3D-printed parts to 30° for the roughing process and to 40° for the entire machining process including finishing.

## 1. Introduction

To improve the surface quality of selective laser molten parts and thus to enable those applications, which require superior surface roughness at least on the technically operating, functional surfaces, tight tolerances [1], restrictive process standards [2,3], or sterilizability in medical applications [4], 3D-printed parts are, generally, post-processed in downstream processes by means of, e.g., turning, milling, or short-blasting, respectively [5,6,7]. In order to reduce the manufacturing efforts in such process chains, different hybrid approaches have been developed that combine additive with subtractive processes [8,9,10,11]. A promising variant of these hybrid approaches combines laser powder bed fusion (LPBF) with in situ milling [12], allowing for the freedom of design offered by additive manufacturing (AM) [13,14] combined with the accuracy and surface quality of precision milling within a single, automated process [15,16]. In particular, precision milling allows for the manufacturing high-value-added parts with high form accuracy and superior surface quality that is generally not attainable by pure LPBF, thus allowing mechanical or biomedical applications [17,18]. The advancement of such hybrid approaches requires a thorough understanding of the fundamental properties of these new technologies, their restrictions and requirements. The specific hybrid approach under investigation in this study has recently been investigated with respect to design rules [19] and fundamental properties [12,20]. In addition, different applications as the generation of medical parts with superior surface roughness and fitting accuracy have been demonstrated [21,22,23,24]. However, the milling process itself has not been particularly addressed, though it significantly differs from conventional milling, as it is performed within the powder bed, i.e., no cooling lubricant is applicable.

Against this background, the authors report on a comprehensive study of the milling process and milling cutter properties of a three-axis milling combined in situ to a conventional LPBF machine with the objective to augment the fundamental knowledge base of this promising hybrid approach. The authors specifically study the tool wear of the milling cutter, determine the average tool life governed by flank wear, and evaluate the accessible surface roughness versus service life of the cutter, aspects that have not been comprehensively reported before. In addition, by optimizing the milling strategy, the maximum accessible milling angle is reduced without deteriorating the surface quality. Overall, the demonstrated quality in terms of surface properties accessible by the virtual layer-wise in situ high-speed milling of a 3D-printed part underlines the practical value of the research.

## 2. Materials and Methods

### 2.1. Machine and Process

**Figure 1:**
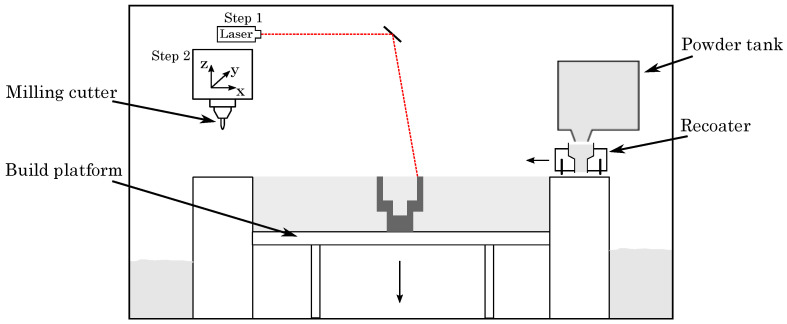
Schematic illustration of the hybrid additive manufacturing unit.

The hybrid additive manufacturing approach, adopted by the Lumex Avance-25 printer (Lumex Avance-25, Matsuura Machinery GmbH, Wiesbaden, Germany), combines laser powder bed fusion and high-speed milling, as schematically illustrated in Figure 1. The LPBF-process (step 1) does not differ from the already well-known standard additive manufacturing approach of selective laser melting (SLM), which has been evaluated comprehensively by various studies [25,26,27]. High speed milling (step 2) is realized by a 3-axis milling system, machining the printed parts after a defined number of built layers (typically, 3–10 layers), i.e., interrupting the LPBF-process as to allow access of the milling cutter to all surfaces, facilitating superior surface roughness also for undercuts and wells with high aspect ratio.

For LPBF, a $P_{L}=500$ W ytterbium fibre laser (SPI Lasers plc, Southampton, Great Britain) with an operating wavelength of λ=1070 nm is used. The laser spot size is set to $d_{\mathrm{spot}}=200\;\mu$m at focus position. To elude deformation by curling due to residual stress, the build platform is heated to $\vartheta_{\mathrm{plate}}=50$ °C permanently, maintaining the machined part at an elevated temperature. The maximum build volume is 250 mm × 250 mm × 185 mm (width × depth × height). As this study focusses on the tool wear and milling characteristics of the high-speed milling process, we applied previously optimized and reported process parameters for the LPBF (cf. Table 1) [20], litigating maraging tool steel 1.2709.

The high-speed milling process operates with a maximum spindle speed of up to 45,000 revolutions per minute and a maximum turning moment of 1.31 Nm. The used milling cutters are solid carbide cutting tools with a nano alloy composed of aluminium, titanium, and silicon for the reduction of wear characteristics (Mitsubishi Materials Corporation GmbH, Meerbusch, Germany). A twentyfold tool magazine allows changing the milling device, e.g., for roughing and finishing purposes, again not differing to industrial high-speed milling machines. Contrary to conventional milling processes, however, no cooling lubricant can be used as milling is performed within the powder bed, posing challenges such as, e.g., elevated temperature conditions or increased tool wear [28,29,30,31]. To access the tool wear properties in our study, we used previously determined milling parameters as given by Table 2 [20].

**Figure 2:**
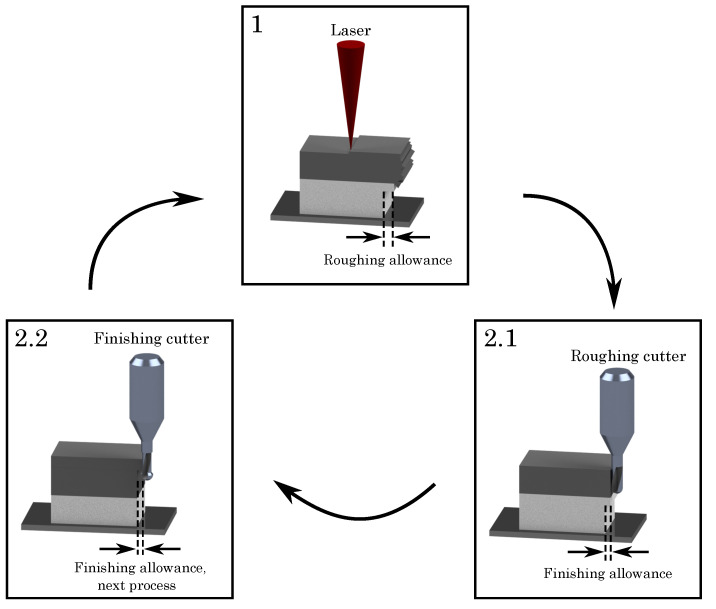
Two-stage milling process with two different milling parts for the hybrid additive manufacturing approach.

For high-speed milling, the LPBF-process is, as mentioned before, interrupted after several layers. Here, we halt after processing ten layers with a height of 50 μm each, a typical value employed before [12,20,32,33]. As shown in Figure 2, a material allowance (150 μm) is introduced by the LPBF-process, being removed gradually during milling using a roughing and a finishing sequence, employing different milling cutters. Firstly, the roughing cutter detaches 120 μm of the material allowance, working downwards the geometry, starting at the last built layer. Secondly, the finishing cutter removes the remaining 30 μm of the material allowance, ensuring best surface quality and final part dimensions.

In contrast to the roughing cutter, the finishing process starts milling from the bottom to the top of the built component. Additionally, the last several layers at the top are spared for the next process cycle, as shown in Figure 2, step 2.2, where the finishing cutter leaves five layers. The reason for this reversed milling strategy is the generated heat input of the LPBF-process, leaving a thermal gradient within the built part with a higher temperature at the last built layers [34,35]. In view of these thermal conditions, it is recommended to start milling at the cooler layers at the bottom and stop milling beneath the upper surface, sparing several hot layers beyond [36,37,38]. As a result, the milling process takes place geometrically shifted, avoiding thermal deformations and ensuring geometrical accuracy and a high surface quality.

**Figure 3:**
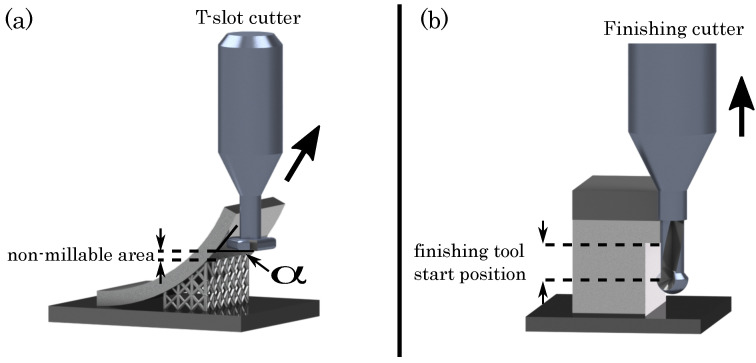
(**a**) Maximum millable angle of the T-slot milling cutter; (**b**) Finishing tool start position.

Moreover, it is worthwhile mentioning that in order to minimize the risk of any damage to the milling cutter, the approach movement of the finishing cutter does not directly target the last spared layers. To avoid unexpected collisions with any protruding material, the finishing tool start position is set apart, in this study 1 mm (cf. Figure 3b). As a consequence, the finishing cutter immerses deeper into the powder bed than necessary, starting milling beneath the last spared layers of the material allowance.

**Figure 4:**
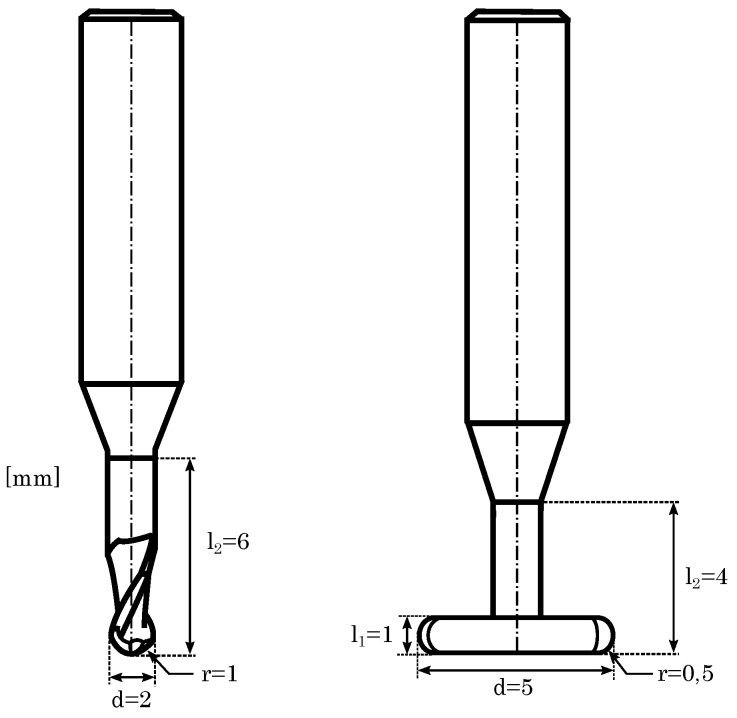
Geometry of the 1 mm ball end mill and the T-slot milling cutter.

In combination with the 3-axis milling system, enabling the milling of inclined structures with the use of T-slot milling cutters (cf. Figure 4), this safety distance limits the machinability of undercut surfaces. The milling system permits, under application of standard process parameters, a postprocessing from an inclination angle of α= 52°, as depicted in Figure 3a. Due to a variation of the process parameters, this maximum inclination angle, in this study, can be optimized, as shown in Section 3.3.

### 2.2. Wear Characteristics and Tool Life

The milling process is a subtractive process including a material removal at the work piece as well as an appearance of wear characteristics at the milling cutter [39,40,41]. Different mechanical, thermal, and chemical impacts are responsible for decreasing tool performance during tool life time [42,43,44]. Most frequently occurring wear causes for milling cutters are adhesion, abrasion and diffusion [45,46]. Due to adhesion, machined material can adhere to the coating of the milling tool. As a result, a built-up cutting edge can develop superposing the main cutting edge and deteriorating its function [47,48]. In addition, the adhered material itself becomes worn and particles of the cutters coating are dissipated, again decreasing the milling quality [49,50]. In turn, abrasion of small, hard particles cause wear at the cutting edge, invading the surface of the milling tool, generating marks and corrugations [51,52]. However, the abrasive particles can derive from the tool- or the cutting-flank of the milling cutter, causing its own tool wear [53]. Finally, diffusion describes the exchange of elements of the cutters coating at elevated tool temperatures [54]. Particular diffusion of hardening elements from the cutters’ surface provokes higher susceptibility to abrasion, in turn reinforcing other wear causes such as adhesion and abrasion [55]. High temperatures induce and accelerate diffusion, since the movability increases, enabling the transition of elements [54]. Within this process, important, hardening elements diffuse from the cutting surface, provoking a higher susceptibility to abrasion. Thus, diffusion mainly reinforces other wear causes such as adhesion and abrasion [55].

Though different wear causes, in general, may lead to different wear characteristics of a milling cutter, in high-speed milling the main wear characteristic of ball end mills is governed by flank wear [56,57,58], which is also observed as the main wear characteristic in our study, while other wear appearances are not identifiable. Noteworthy, crater wear appears as the second most common wear characteristic at high cutting speeds, occurring at low feed rates [59].

**Figure 5:**
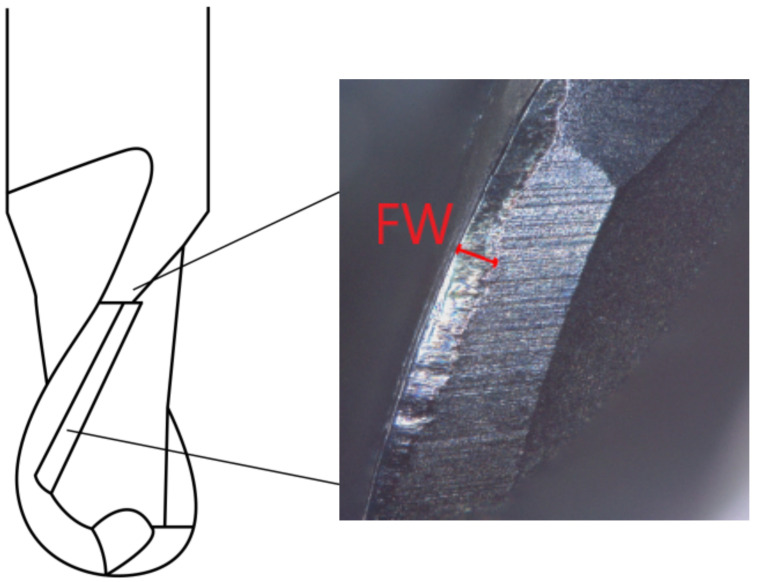
Illustration of the flank wear (FW) on a ball mill.

Flank wear occurs at the cutting edge of the ball end mill. As depicted in Figure 5, the material becomes dissipated perpendicularly to the cutting edge, based on adhesion and abrasion, and might be reinforced by diffusion. The latter occurs as a result of high temperatures during processing, which has to be specifically considered in our study, as milling takes place within the powder bed, i.e., no cooling lubricant can be used [42]. Thus, the cutting edge becomes dull and the strength of the milling cutter decreases, increasing the probability of a tool breakage [60]. Additionally, the surface quality and the geometrical accuracy decline with a dull cutting edge [61,62].

Thus, methodically, the flank wear is used to quantify tool wear and to define milling tool life criteria. As the material removal at the cutting edge of the ball end mill occurs perpendicularly to the cutting edge, the width of the removed area is measured in equal direction and named as FW (Figure 5).

**Figure 6:**
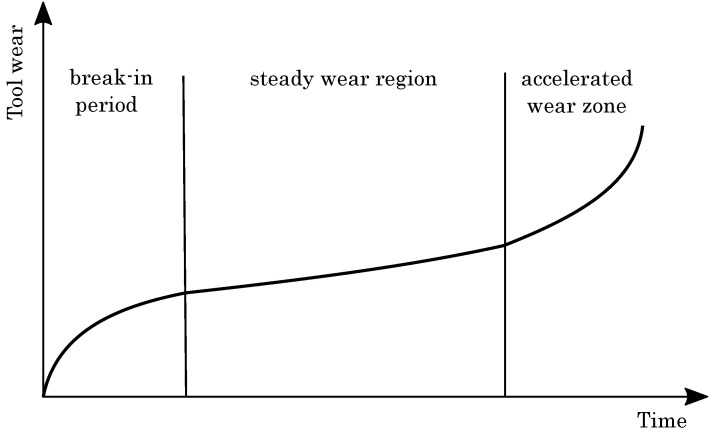
Typical wear evolution in dependence on milling time.

As a milling tool life criterion, the average flank wear level can define the maximum usage time for milling cutters. Figure 6 depicts by trend the average flank wear as a function of time, revealing the typical s-shaped dependence. At first, the primary wear zone or break-in period with a rapid initial increase of the FW reveals, induced by the direct blunting of recently sharpened cutting edges. The rapid growth quickly flattens after the milling cutter develops its work sharpness and the steady wear region (secondary wear zone) with its uniform wear rate following [63]. Finally, the milling cutter accomplishes the failure region (tertiary or accelerated wear zone), as being characterized by a final sharp increase of FW until the final failure occurs [64,65,66].

### 2.3. Optical Characterization Tools

Different optical characterization tools are used to measure the surface roughness and the average flank wear. In particular, the flank wear of the milling cutter is determined by digital microscope DVM6 (Leica Microsystems GmbH, Wetzlar, Germany). The used lens is a PlanAPO FOV 12.55 with a maximum zoom of 675:1. The FW is captured at the same point of the milling cutter with several images, using the z-stack function, to obtain the best results and to generate a good depth of field. To avoid light reflections, a diffuser is used. Every flank was measured at 15 positions at least to ensure statistically reliable analysis.

The roughness, in detail, the average surface finish Ra and the maximum height Rz of the LPBF fabricated and milled surfaces, is measured by laser scanning microscope VK-X200 (Keyence GmbH, Neu-Isenburg, Germany). The approach follows ISO 4288 for geometric product specifications—surface textures, according to which the measurement is executed above 4.8 mm and the L-Filter is set to $\lambda_{C}$ = 0.8 mm. Pictures taken with 20× magnification are analyzed with the VK-Analysis Module (Keyence GmbH, Neu-Isenburg, Germany). Every measurement was taken thrice by the use of two sets of specimens.

## 3. Results and Discussion

### 3.1. Flank Wear

**Figure 7:**
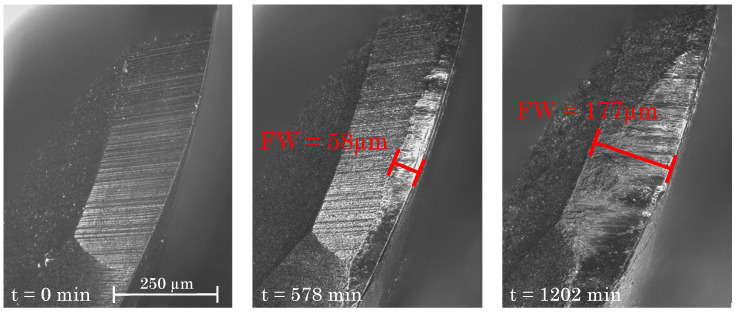
Development of the flank wear at different milling times.

The flank wear at the cutting edge exhibits the highest peculiarity at the position where the milling cutter enters the material. In the direction towards top of the cutting tool, FW becomes smaller. For a reliable analysis, the FW is always measured at the maximum point for several times, specifying the average value as FW${}_{max}$. Figure 7 depicts the development of the flank wear of the milling tool, exhibiting maximum at the main cutting edge and exposing the measurement perpendicularly to the cutting direction. Specifically, Figure 7 shows the temporal evolution of the flank wear beginning at the start of the milling process (t = 0 min) up to t = 1202 min, for which a pronounced flank wear of 177 μm is determined.

### 3.2. Evaluation of Tool Life in Correlation with Surface Roughness

**Figure 8:**
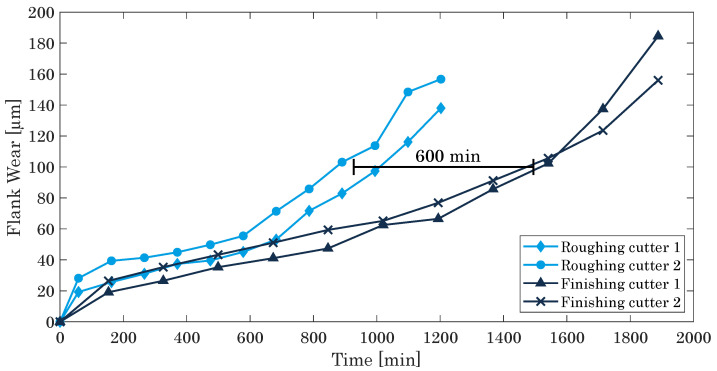
Comparison of the flank wear of two different roughing and finishing cutters.

The tool life of the roughing and the finishing cutter differs strongly, depending on the proceeding of the two different milling steps. The higher load, the roughing cutter faces during the process, causes this difference. As outlined in Section 2.1, the roughing cutter removes 120 μm of the material allowance, while the finishing cutter only ablates 30 μm. Thus, the FW of the roughing cutter rises more pronounced than the FW of the finishing cutter. For the comparison of the two kinds of milling cutters, the FW of the roughing and the finishing cutter are depicted as a function of time in Figure 8 for two cutters each. For all cutters, the characteristic s-shaped trend of the flank wear is found, as discussed in Section 2.2, showing a pronounced break-in period within the first 50 min (roughing cutter) and 150 min (finishing cutter), an extended secondary wear zone with a uniform wear rate up to 900 min (roughing cutter) and up to about 1300 min (finishing cutter), and finally followed by an accelerated wear zone as characterized by a sharp increase for milling durations above 950 min (roughing cutter) and about 1600 min (finishing cutter). Seemingly, the cutting edge of the roughing tool becomes more worn, increasing the FW directly and leading to an offset of 600 min at the critical point of the FW at 100 μm.

**Figure 9:**
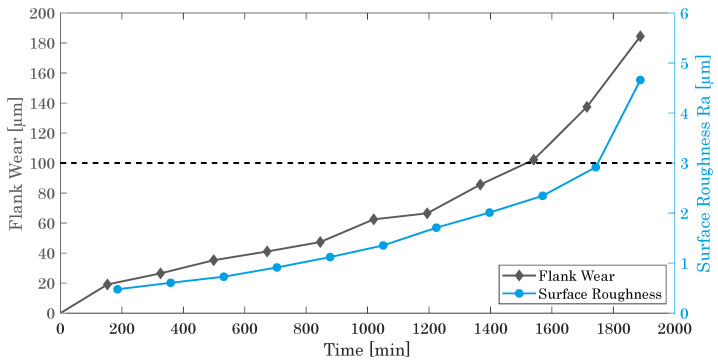
Flank wear of the finishing cutter and surface roughness as a function of time.

However, the overall evolvement of the FW for both milling cutters is very similar, revealing the typical s-shaped dependence. At closer examination, Figure 9 depicts the FW of the finishing cutter as a function of time as well as the applied maximum limit of 100 μm. Additionally, the surface roughness, in detail, the average surface finish Ra, is depicted at the secondary y-axis.

Apparently, the typical s-shaped dependency starts with a rapid initial increase of the FW within the first 150 min of tool operation, representing the primary wear zone or break-in period. Afterwards, the steady-state wear region is reached (secondary wear zone), characterized by a uniform wear rate of up to about 1300 min of operation [45]. Finally, the milling cutter accomplishes the accelerated wear zone with its final sharp increase of the FW until the final failure occurs [64,65,66]. For the cutting tool under investigation, we identify this regime after about 1900 min of operation with an associated FW of about 180 μm (Figure 9).

In general, tools with an extended secondary wear zone with low wear rate are preferential, guaranteeing a longer tool life, better milled surfaces, higher repeatability, and an overall robust milling process. As Figure 9 shows, the roughness increases similarly as the FW, yet with a sharper increase after about 1750 min of operation. In conjunction of FW and Ra and in accordance with a typically accepted FW limit of 100 μm (dashed line in Figure 9), we consider the milling cutter to properly work for about 1550 min. Within this service life, the generated roughness of maximal Ra = 2.5μm is in the range of industrial applications of milling processes with Ra = 1.6–12.5μm [67,68,69].

In addition to the evaluation of the tool life progress, recommended tool life sections were defined with regard to the accessible surface quality. Thus, the machine life of the milling cutter can be determined for a selected range of surface quality of the fabricated component. As shown in Table 3, the sections are structured according to the surface roughness Ra with steps of 0.3 μm.

### 3.3. Optimization of the Maximum Millable Angle

The machinability of undercut surfaces of the components is, with regard to the 3-axis milling system and the usage of T-slot cutters for undercut parts, limited to a maximum millable angle of 52°, applying standard process parameters. This maximum angle is primarily defined by preexisting factors as the geometry of the T-slot milling cutter and secondly by parameters, concerning the milling process itself, e.g., the start position of the finishing cutter and the number of the afore-built layers.

In the following examination, the variable process parameters will be customized, enlarging the process window for the maximum millable angle of the milling system. Therefore, the number of the built layers between the consecutive milling process will be reduced; consequently, the milling process is used more frequently as well as the finishing tool start position being varied. As a result, the process duration will rise and the surface quality might be affected negatively by thermal effects. The consequences will be monitored with the evaluation of the surface quality, comparing the values of the average surface roughness Ra as well as the maximum height Rz with the surface quality of specimens, manufactured with standard process parameters. Additionally, the surface quality will be evaluated as a function of the inclination angle for a general classification with respect to existing literature.

#### 3.3.1. Milling Process for Application of Standard Process Parameters

The milling process proceeds, as declared comprehensively in Section 2.1, with an initiation of the milling cutters, interrupting the LPBF-process. Using standard process parameters, 10 layers with 50 μm height each are built, before the milling cutter starts. Further, the finishing cutter maintains a safe distance of 1 mm, increasing the cooling time for the last exposed layers. Thus, the finishing process starts at layers built two process cycles before, embracing 10 layers each.

**Figure 10:**
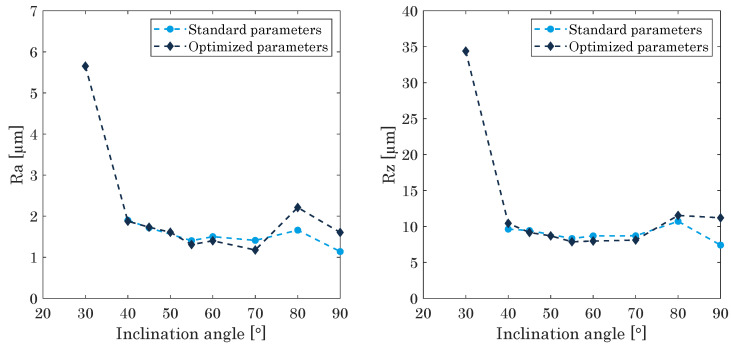
Surface roughness of the inclined structures for the application of standard and optimized process parameters.

In previous research, it was evaluated that specimens with an inclination angle lower 30° cannot be machined by the 3-axis milling system. Inclinations between 40°–52° can only be milled by the roughing cutter, exhibiting a higher value for the surface roughness (cf. Figure 10, Ra = 1.5 μm–2 μm) and displaying slight discolouration. Inclination angles greater than 52° can be machined entirely, improving the surface quality to Ra < 1.5 μm, to keep up with conventional milling systems.

#### 3.3.2. Milling Process for Optimized Parameters

For the optimization of the maximum millable inclination angle, both variable parameters, the start position of the finishing cutter and the number of afore-built layers, are varied. The number of the afore built layers decreases and the tool start position for the finishing cutter is adjusted, maintaining that the first machined surfaces were built two process cycles before, as mentioned in Section 2.1. However, in order to avoid an additional moving of the recoater to the home position before milling, the number of afore-built layers has to be even.

Within this study, the number of afore-built layers is set from ten to six, accordingly, 300 μm of the components are built, before the milling process begins. The start position for the finishing cutter is adjusted to 0.5 mm. Please note, the reduction to eight layers does not significantly increase the performance of the T-slot cutter. In turn, a decrease to four or two layers does affect the maximum millable angle significantly, but also rises the processing time extremely, e.g., by four times for the setting of two built layers.

Under application of the adjusted process parameters, it was possible to reduce the maximum millable angle, manufacturing the specimens reliably. Inclinations with an angle lower than 20° are still not machinable, while inclinations of up to 39° can be milled by the roughing cutter, exhibiting a slight discolouration and an increased surface roughness compared to completely milled surfaces. Specimens with an angle larger than 40° can be milled completely, exhibiting a less pronounced discolouration than the roughed ones and excluding a thermal distortion. The surface roughness for the completely machined specimens does not exceed the surface quality of the standard built components, as depicted in Figure 10. Thus, the modulation of the process parameters does not affect the quality of the components negatively, expanding the process window for the maximum millable inclination angle, as summarized in Table 4.

## 4. Conclusions

The hybrid additive manufacturing approach, encompassing laser powder bed fusion and high-speed milling has been evaluated with respect to tool wear, milling characteristics, and achievable maximum millable angle for undercut surfaces of 3D-printed parts.

For the milling process taking place inside the metal powder bed, thus constituting a dry machining approach, flank wear is identified as the primary tool wear characteristic. In addition, it has been shown that by virtue of the greater load, the tool life of the roughing cutter is lower than the tool life of the finishing cutter (difference at FW = 100 μm: 600 min).

However, the typical s-shaped dependence for the tool life of the milling cutters was confirmed and the maximum limit of 100 μm for the FW delimits the fabrication with a good surface quality. Additionally, recommended tool life sections were defined for selected surface roughness areas. Thus, the tool life of the milling cutters can be chosen with respect to the desired surface roughness for the manufactured components.

The milling process itself, with its specific properties and limiting factors with regard to the manufacturing of undercut surfaces, originating from the 3-axis milling system, was investigated. The maximum millable angle for inclined structures was reduced to 30° for the roughing cutter and to 40° for the finishing process. Therefore, the finishing tool start position and the number of built layers between the processes have been decreased, exhibiting no negative consequences for the built component. The surface roughness was defined as a function of the inclination angle, leading to approximately Ra = 1.5–2.0 μm and Rz = 7.5–12 μm for finished undercut surfaces, increasing strongly to approximately Ra = 5.5 μm and Rz = 35 μm for roughed only surfaces. Overall, this contribution outlines previously unreported, yet fundamentally and practically relevant, properties of the virtually layer-wise in situ milling process for hybrid additive manufacturing, highlighting superior surface properties allowing for the manufacturing of high-value-added parts with high form accuracy.

## Figures and Tables

**Table 1:** Process parameters of LPBF.

	Laser Power [W]	Scan Speed [mm/s]	Hatch Distance [μm]
Area	320	700	0.12
Contour	320	1400	—
Support	320	700	0.12

**Table 2:** Milling process parameters.

	Z-Pitch [mm]	Spindle Speed [rot/min]	Feed Rate [mm/min]
Roughing cutter	0.15	30,000	2000
End mill	0.1	30,000	1600

**Table 3:** Recommended tool life sections for selected surface roughness.

Surface Roughness		Tool Life	
Rz [μm]	Ra [μm]	Roughing [min]	Finishing [min]
>5	>0.7	260	180
>7	>1.0	710	530
>9	>1.3	1160	880
>10	>1.6	1350	1000
>14	>1.9	1610	1220
>15	>2.3	2020	1540

**Table 4:** Machinability of inclined structures as a function of inclination angle. Legend: X: not millable, (*√*): roughed, *√*: completely machined.

Inclination	10°	20°	30°	40°	45°	50°	55°	60°	70°	80°	90°
Standard	X	X	X	(*√*)	(*√*)	(*√*)	*√*	*√*	*√*	*√*	*√*
Optimized	X	X	(*√*)	*√*	*√*	*√*	*√*	*√*	*√*	*√*	*√*
